# Time-course of oral toxicity to contaminated groundwater in male Sprague Dawley rats

**DOI:** 10.1016/j.toxrep.2024.05.002

**Published:** 2024-05-17

**Authors:** Bright Boamah, Steven Siciliano, Natacha Hogan, Markus Hecker, Mark Hanson, Patrick Campbell, Rachel Peters, Ahmad N. Al-Dissi, Lynn Weber

**Affiliations:** a Toxicology Centre, University of Saskatchewan, Saskatoon, SK, Canada; b Environment and Geography, University of Manitoba, Winnipeg, MB, Canada; c WSP Canada Inc., Winnipeg, MB, Canada; d Federated Co-operatives Limited, Saskatoon, SK, Canada; e Veterinary Pathology, Western College of Veterinary Medicine, University of Saskatchewan, Saskatoon, SK, Canada

**Keywords:** Complex mixture, Target organ, Time course, Effects directed toxicity testing

## Abstract

Assessing toxicity of complex mixtures of contaminants from industrial sites with historic and ongoing contamination remains a challenge for risk assessors. Groundwater from a pesticide packaging site in Canada containing a complex mixture of known and unknown contaminants was examined in male rats to determine the target organ toxicity. This study determined the time-course of toxicity (7, 14, 28, and 60 days) following *ad libitum* oral exposure to 0.05% v/v contaminated groundwater compared to tap water (control) in male Sprague Dawley rats (n=5 /group/time). Exposure to groundwater resulted in inflammation, indicated by a statistically significant increase in plasma lymphocyte and neutrophil counts on days 7 and 60, respectively, but a reduction in the plasma alpha 2 macroglobulin levels by day 60. Gonadotoxicity was indicated by a reduced Johnsen score (grading spermatogenesis) in all exposed groups at all time points, while seminiferous epithelial height was reduced on days 7, 14, and 28 compared to controls. Plasma testosterone was reduced in exposed groups on days 7 and 28, accompanied by elevated testicular lipid peroxidation at all time points compared to control. In contrast, lipid peroxidation in the lungs from exposed rats was elevated on days 7, 14, and 28. Plasma symmetric dimethylarginine was elevated on day 14 in the exposed group indicating renal impairment. Taken together, these results indicate that testes, kidney, immune and lung are target organs for the contaminated groundwater from this industrial site. The current study highlights the challenge in hazard assessment for complex mixtures and highlights the need for effects-directed analysis and the continued, albeit limited, use of animal models in toxicity testing.

## Highlights

• Effects-directed approach used to determine toxicity of a complex groundwater in a rat model.
• Water from an industrial site in Canada with known and unknown contamination was tested.
• Testes, kidney, immune and lung were target organs and greater than predicted by known contaminants.
• Results support the use of effect-directed toxicity testing when unknown contaminants are present.

## Graphical Abstract

**Figure:**
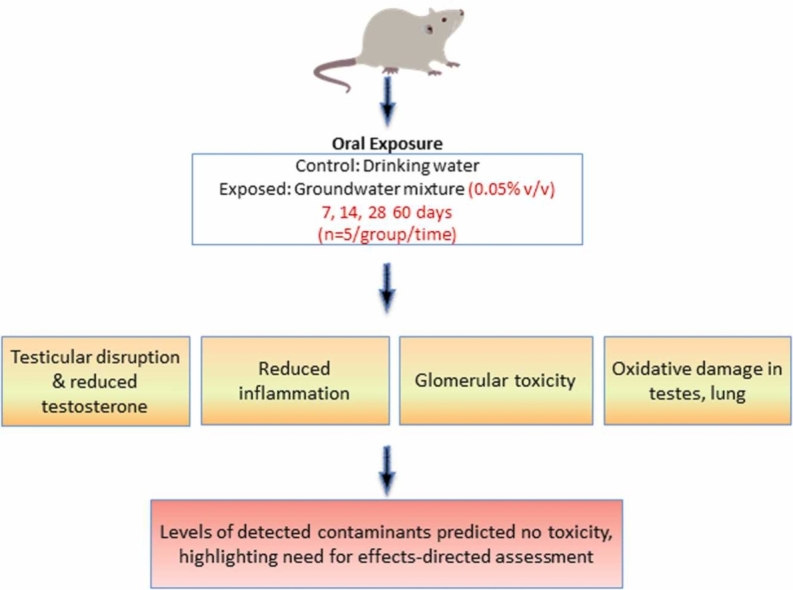

## 1 Introduction

Chemicals are an indispensable part of human and ecological life; however, the presence of these compounds in the environment poses inherent toxicological and environmental risks. Humans are predominantly exposed to multiple chemical constituents at different concentrations and routes instead of single compounds at fixed concentrations [28]. Previously, the approach to human health risk assessment following exposure to these complex mixtures was based on the evaluation of the individual components (component-based approach or CBA) of the mixture [9]. However, the assumptions of the component-based approach for complex mixture exposure lead to overestimation or underestimation of the toxicity induced by the constituents of the mixture [21]. The whole mixture approach improves upon CBA incorporation of the adverse effects posed by chemical-chemical interactions and metabolites within the mixture [3]. Activities at complex legacy sites can lead to extensive, persistent, and continuous environmental contamination with toxicants, including petrochemicals, pesticides, heavy metals, and other emerging chemicals [15]. In this study, we identified contaminants (pesticides, petroleum hydrocarbons and heavy metals) in groundwater samples from an industrial site. Unfortunately, in legacy-contaminated sites, human and ecological risk assessment methods are limited by their inability to predict the adverse effects of dynamic mixtures involving not just parent compounds, but also metabolites and unknown compounds that may not be detected even in the most comprehensive chemical analyses [31]. In the last decade, significant improvements in the identification of individual compounds in complex mixtures have been observed; however, a major challenge in relating the identified constituents as drivers of target organ toxicity still exists.

In this study, a legacy-contaminated site from an active pesticide packaging facility within an urban area was used for other industrial activities that date back to the 1950 s. A recent study from this group using extracts of contaminated soil from this same industrial site identified a pattern of immunostimulatory effects and nephrotoxicity along with evidence of exposure to aryl hydrocarbon agonists and metabolites of cholinesterase inhibitors [4]. The biological processes underlying target organ effects suggest that petroleum hydrocarbons and pesticide breakdown products are potential drivers of soil contaminant toxicity [4]. Although almost all organs were screened in a previous study using soil extracts, no toxicity assessment was performed in rats following oral exposure to groundwater from the same site.

There is a high potential for unknown compounds in this study site’s groundwater, despite extensive chemical analyses. Therefore, this study aimed to identify the target organs, biological processes, and likely drivers of toxicity using an effects-directed approach in a rodent model that could be used in subsequent human health toxicological risk assessment. We hypothesized that oral exposure to low-dose contaminated groundwater from this study site would induce similar target organ toxicity in male Sprague-Dawley rats. A previous toxicity study from our group in Sprague Dawley rats following oral exposure to soil extracts found immunostimulatory and nephrotoxic effects in a pattern consistent with petroleum hydrocarbons and cholinesterase pesticides [4]. In this study, biochemical and histopathological target organ toxicities were measured in male Sprague Dawley rats (n=5/group/time) orally exposed to a 0.05% v/v groundwater mixture for 7, 14, 28, and 60 days.

## 2 Materials and methods

### 2.1 Collection and chemical analysis of groundwater from study site

Groundwater samples were collected from high-impact zone wells at a pesticide packaging industrial site in Canada at depths ranging from 0.63 to 5.37 m below ground level during the fall season of 2020 (see Figure S1 for a site map). The label of ‘high impact’ was based on the proximity of the wells in the facility to areas where high industrial activities (packing, mixing, and storage of pesticides) occurred. Samples were equally mixed for a given well and stored in the dark at temperatures ranging from 4 to 6 °C until use. The proximity of the facility to a railway also increases the risk of petroleum contamination. A previous study by Boamah et al. [4] identified two out of three most toxic soil sample sites, as determined by biochemical and histopathological alterations in target organs, to be located a few meters from the high impact well used in the current study. Full chemical characterization of the groundwater samples collected in Fall 2020 and used in the current study are shown (Supplemental Tables S1 and S2). Comparing to previous and subsequent analyses of groundwater from the same well (2019 and 2021) show a fairly consistent composition to the contamination. For comparison, the groundwater collection from the same high impact groundwater well 6 months later (in June 2021) showed similar contaminants of concern (see HIAZ well in supplemental Table A5 of [11]). Specifically, the chemical compounds in groundwater samples collected from the high impact areas of the study site in 2020 and used in the current study were characterized with gas chromatography/mass spectrometry (GC-MS) or liquid chromatography with tandem mass spectrometry (LC/MS-MS) for petroleum hydrocarbons and pesticides, while and the collision/reaction cell with inductively coupled plasma mass spectrometry (CRC-ICP-MS) was used to identify heavy metals.

### 2.2 Experimental model

Sprague Dawley rats of 150–200 g were acquired from the Charles River Laboratories (Senneville, Quebec, Canada). Thirteen male Sprague Dawley rats were used for the range-finder study, and another 40 male Sprague Dawley rats were used for the main experiment. The rats were placed in individual cages for the range-finder and main experiments. For each set, the rats were acclimatized for 7 days at a temperature of 22°C, ambient humidity and a 12-hour light/dark cycle upon receipt at the Animal Care Unit at the Western College of Veterinary Medicine (Saskatoon, SK). During the acclimation phase and the main experiment, the rats were provided water and a standard rat maintenance diet (Prolab RMH 3000, Missouri, USA) *ad libitum*. All experiments were approved by the Animal Research Ethics Board at the University of Saskatchewan according to the guidelines of the Canadian Council on Animal Care under an animal use protocol (20170109).

### 2.3 Range-finder experiment

Thirteen male Sprague Dawley rats were placed into four different groups (control and three groundwater exposure groups) for 7 days. The control group (n= 4 rats) was provided tap water (City of Saskatoon, SK, Canada), and the exposed group was provided contaminated groundwater (high impact) at 0.05%, 0.5%, and 5% v/v (n=3 rats/exposed group). Multiple endpoints were assessed at the end of the study to determine the appropriate concentration of high impact groundwater for use in the main time-course study. The endpoints included health assessments (body weight, organ weight, food intake, water intake, urine output, and general appearance), renal function (blood urea, creatinine, electrolytes, and creatinine clearance), liver function (aspartate transaminase, alanine transaminase, albumin, globulin, total bilirubin, and alkaline phosphatase), plasma cholesterol, and electrocardiography (P duration, heart rate, QRS complex duration, and QTc duration).

### 2.4 Time-course experimental protocol

Forty Sprague Dawley rats were randomly divided into four treatment groups that were exposed to groundwater for 7, 14, 28, and 60 days, with each group comprising of 10 male rats (5 controls + 5 exposed). To ensure sub-lethal responses, a preliminary range-finder experiment was conducted, and 0.05% v/v was determined to be sub-lethal after 7 days of oral exposure via drinking water (see Supplemental Material). Each group was provided *ad libitum* with either drinking water (control group which used tap water) or contaminated groundwater (0.05% v/v exposed groups)*.* Dosing concentrations were prepared weekly by diluting the collected groundwater samples in tap water and storing them in the dark at temperatures ranging between 5 ± 1°C. Daily monitoring of overt clinical health variables (behavior, appearance, and grooming) was performed using a rodent-specific health assessment checklist [5] during the course of the study.

### 2.5 Sample collection and basic toxicological measurements

Body weight, feed, and water intake were measured at each time point at baseline and subsequently every week until the end of the study period (days 7, 14, 28, and 60). Urine output was measured (metabolic cages) on day 0 and on the final day for each time point. Blood was collected via cardiac puncture into serum EDTA tubes, followed by an overdose of isoflurane at the end of each specific time point. Plasma was collected by centrifugation (3000 x g for 12 mins), aliquoted, and frozen at −80°C prior to analyses. At the end of each time point, the rats were euthanized, and the organ weights of the kidney, liver, brain, spleen, testes, lungs, and heart were measured. Organ-to-body-weight ratios (relative organ weights) were calculated and expressed as mg/g. Portions of the organs were fixed for histological analyses, frozen in liquid nitrogen, and stored at −80 °C until biochemical analyses were performed.

### 2.6 Assay of the oxidative stress biomarker

Oxidative stress was measured in the liver, lung, kidney, and testis using a thiobarbituric acid reactive substances (TBARS) assay kit, which measures the levels of malondialdehyde (MDA; Bioassays Systems, Hayward, USA). Each tissue sample (liver, lung, kidney, and testes) of 100 mg was weighed and homogenized in 200 μL of phosphate-buffered saline (pH=7.4). Subsequently, 75 μL of each sample was added to 150 μL of trichloroacetic acid and centrifuged at 12,900 x g for 5 minutes at 4°C. The supernatant was used for biochemical analyses as previously described [26].

### 2.7 Assessment of plasma inflammation

Alpha-2 macroglobulin ELISA kit (Cell Bio Labs, San Diego, CA, USA) was used to measure plasma inflammation. The standards and samples were added to each well (50 μL) and incubated for two hours. The plate was washed with 1X wash buffer, followed by the addition of 50 μL biotinylated antibody per well. The standards and samples in the wells were incubated for 1 hour. The plate was then washed again with 1X wash buffer, and 50 μL of chromogen substrate was added to each well. The samples were incubated for 10 min at room temperature. Finally, 50 μL of stop solution was added and the absorbance was read at 450 nm using a microplate reader (SpectraMax ABS Plus, Molecular Devices Corp.).

### 2.8 Symmetric dimethylarginine assay

A plasma symmetric dimethylarginine (SDMA) ELISA kit (MyBioSource Inc., San Diego, CA, USA) was used to determine glomerular toxicity. Plasma samples and standards (40 μL) were added to respective wells, followed by the addition of 10 μL anti-SDMA antibody. Streptavidin-HRP (50 μL) was added to plasma samples and standards, followed by an incubation period of 60 minutes at 37°C. Washing of the plate using 1X wash buffer was done followed by the addition of substrates A and B (50 μL each), which were subsequently incubated for 10 minutes at 37°C in the dark. A stop solution (50 μL) was added to the plates and the absorbance was read at 450 nm using a microplate reader (SpectraMax ABS Plus, Molecular Devices Corp.).

### 2.9 Testosterone assay

Plasma was ether-extracted and resuspended in a gelatin-containing buffer, then frozen until assay as previously described [6]. Testosterone levels were determined in ether-extracted samples using an ELISA kit (Abnova, Taipei, Taiwan). Standards, samples, and reagents were prepared in accordance with the manufacturer’s instructions. The samples and standards (10 μL) were added to the appropriate wells, and 100 μL of the buffer solution was added. An additional 50 μL of the enzyme conjugate was added followed by a 60-minute incubation duration at room temperature. The plate was washed using the required buffer, followed by addition of 200 μL of the substrate solution. A final incubation of 30 minutes was performed in the dark and the reaction halted using 50 μL of stop solution. The absorbance was read at 450 nm using a microplate reader (SpectraMax ABS Plus, Molecular Devices Corp.).

### 2.10 Echocardiogram analyses

At the end of each time point in either the range-finder (at the end of 7 days) or in the time-course experiment (7, 14, 28, and 60 days), electrocardiogram (ECG) measurements were performed in rats under isoflurane anesthesia (5% induction, 1–3% maintenance with 100% oxygen) to assess cardio-electrophysiological activity using the PowerLab system and LabChart7Pro [33]. A ten-minute ECG was recorded for each rat after maintenance anesthesia was reached. During the analyses, LabChart7Pro software was used to set profiles specific to rats. Readings were considered after a minute under anesthesia to achieve a stable reading. Nine stable readings (constant baseline) were averaged from the ECG for the considered parameters (QTc interval, PR duration, QRS complex, and heart rate). After ECG recordings, the rats were euthanized with an overdose of isoflurane.

### 2.11 Histopathology

Following euthanasia, the liver, kidney, heart, spleen, testes, and lungs were fixed in 10% formalin for 24 hours and stored in 70% ethanol. Before sectioning at 4-micron thick with an automated microtome (Microm-HM350), organs were processed in a tissue processor (RVG/1 Histology Vacuum Tissue Processor) and embedded in Paraplast Xtra paraffin wax (Leica Biosystems). The sections were stained with hematoxylin and eosin. For histopathological analyses, the slides of all exposed and control groups were evaluated blindly and photographed using a light microscope with a digital camera (Olympus, Japan). The testicular histopathology and spermatogenesis were determined using the Johnsen scoring system [30]. An average of 20–25 seminiferous tubular sections from each rat were graded and scored from 1 to 10, based on the presence or absence of various cells at each stage during spermatogenesis. A high Johnsen score indicates effective spermatogenesis, whereas a low score indicates spermatogenic cell line damage.

### 2.12 Data evaluation and statistical analyses

Statistical analyses were performed using GraphPad software (version 10), and the results were expressed as the mean ± standard error of the mean (SEM). Normality tests were performed using the Shapiro-Wilk analysis. Differences between the control and exposed groups at different time points were estimated using unpaired Student’s t-test with Welch correction. Multiple time points were analyzed using one-way ANOVA and Dunnett’s post-hoc test. Statistical significance was set at P ≤ 0.05.

## 3 Results

### 3.1 Groundwater contamination

Full chemical analyses for organic and inorganic contaminants in groundwater collected from the high impact well during Fall 2020 and used in the current experiment (Tables S1 and S2) indicated that several contaminants were above regulatory (Material Safety Datasheet, Alberta Tier 1, and Ministry of the Environment of Ontario) levels (Table 1). The contaminants of concern included the low molecular weight petroleum fraction (F2), pesticides such as bromoxynil, picloram, 2,4-dichlorophenoxyacetic acid (2,4-D), and dicamba, as well as some heavy metals, including manganese and uranium (Table 1).

**Table 1:** Contaminants of potential concern (COPC) that exceeded guidelines in the groundwater collected from the high impact well during Fall 2020 and used in the current study’s oral exposure.

Contaminants of potential concern	Type of contaminants of potential concern	Maximum concentration in undiluted groundwater (mg/L)	Guideline value (mg/L)	Guideline source
Bromoxynil	Nitrile herbicide	0.161	0.04	Alberta Tier 1 (2019)
Picloram	Herbicide	0.0783	0.029	Alberta Tier 1 (2019)
2,4-D	Phenoxy acid herbicide	0.07	0.004	Alberta Tier 1 (2019)
Dicamba	Benzoic acid herbicide	0.194	0.01	Alberta Tier 1 (2019)
F2	Petroleum hydrocarbon	11	3.1	MOE (2016)
Manganese (Dissolved)	Heavy metal	0.242	0.05	MSD
Uranium	Heavy metal	0.0255	0.015	MSD

F2 = the second lowest molecular weight fraction of petroleum hydrocarbons (e.g., larger than BTEX or benzene, toluene, ethylbenzene, and xylene); 2,4-D = 2,4-dichloro phenoxy acetic acid. Material safety data (MSD) and Ministry of the Environment (MOE) of Ontario.

### 3.2 Range-finder experiment

At the end of the range-finder study on day 7 using groundwater samples collected in Fall 2020, no gross or qualitative signs of toxicity were observed, and no statistically significant differences in body weight or organ weight were observed among treatments or compared to the control group (Table S3). Moreover, no statistically significant changes in biochemical parameters, such as renal function tests, liver function tests, plasma electrolytes, glucose, cholesterol (Table S4), or creatinine clearance (Figure S2), were observed between the control and exposed groups. In contrast, cardiotoxicity in the form of prolonged QTc interval (0.05% high impact), prolonged PR interval (0.5%), and elevated heart rate (0.05% high impact and 0.5% high impact) was observed in the exposed groups when compared to the control group (Figure S3). Based on the evidence of cardiotoxicity, even at the lowest concentration tested in this range-finder experiment, an exposure concentration of 0.05% was chosen for the subsequent time-course experiment to ensure that the exposure was sublethal.

### 3.3 Time-course experiment: mortalities, body weight, organ weight, water and food intake, urine output, and cardiac function

Oral exposure to 0.05% v/v contaminated groundwater at any specific time point did not cause treatment-related mortalities or overt toxicities (no poor grooming and behavioral changes) during the experiment. After 14 days of drinking water exposure, the exposed group showed a statistically significant reduction in liver weight compared with the control group (Table 2). However, no other significant changes in liver weight were observed at any other time point in this experiment. Moreover, there were no significant changes with treatment at any time for body weight, relative organ weights (kidney, liver, lung, brain, testes, spleen, or heart), water consumption, food intake, and urine output (Tables 2 and S5). The body weight, food intake, water intake, and urine output of all rats were within normal reference limits with respect to age [16]. No significant changes in electrocardiogram parameters indicative of arrhythmias were observed among the treatments at the different time points (Figure S4).

**Table 2:** Time-course of 0.05% (v/v) contaminated groundwater in drinking water effects on body and organ weighs in male Sprague Dawley rats.

**Relative organ weight (x10**^**−3**^**g/kg of body weight)**	**Day 7**		**Day 14**		**Day 28**		**Day 60**	
	**Control**	**Exposed**	**Control**	**Exposed**	**Control**	**Exposed**	**Control**	**Exposed**
Kidney	6.84 ± 0.18	6.72 ± 0.15	7.20 ± 0.18	6.80 ± 0.01	5.31 ± 0.32	5.29 ± 0.29	5.77 ± 0.25	5.71 ± 0.19
Liver	38.7 ± 1.02	37.90 ± 0.85	36.69 ± 0.93	34.65 ± 0.42*	33.13 ± 1.99	33.01 ± 1.96	31.45 ± 1.58	31.60 ± 1.45
Lung	7.01 ± 0.18	6.88 ± 0.15	5.03 ± 0.13	4.75 ± 0.01	3.82 ± 0.23	3.81 ± 0.23	4.05 ± 0.18	4.04 ± 0.13
Brain	5.54 ± 0.15	5.44 ± 0.12	5.03 ± 0.17	4.81 ± 0.01	4.74 ± 0.29	4.72 ± 0.28	4.12 ± 0.20	4.13 ± 0.14
Testes	10.23 ± 0.27	10.04 ± 0.23	8.54 ± 0.22	8.07 ± 0.01	7.92 ± 0.48	7.89 ± 0.47	7.67 ± 0.33	7.57 ± 0.25
Spleen	2.58 ± 0.01	2.53 ± 0.01	1.97 ± 0.01	1.87 ± 0.02	1.32 ± 0.01	1.32 ± 0.01	1.79 ± 0.15	1.95 ± 0.01
Heart	3.70 ± 0.01	3.63 ± 0.01	3.16 ± 0.01	2.99 ± 0.01	3.08 ± 0.19	3.07 ± 0.18	3.32 ± 0.17	3.35 ± 0.11
Final Bodyweight (g)	334.6 ± 9.2	340.6 ± 7.2	352.4 ± 3.5	372.4 ± 4.5	409.2 ± 25.1	411.4 ± 28.6	481.3 ± 25.5	473.4 ± 16.0

Data are presented as mean ± SE (n= 5 rats/group). Asterisk denotes statistical significance from control (P < 0.05; unpaired t-test, Welch correction).

### 3.4 Time-course experiment: clinical hematology, systemic inflammation and liver histology

**Fig. 1:**
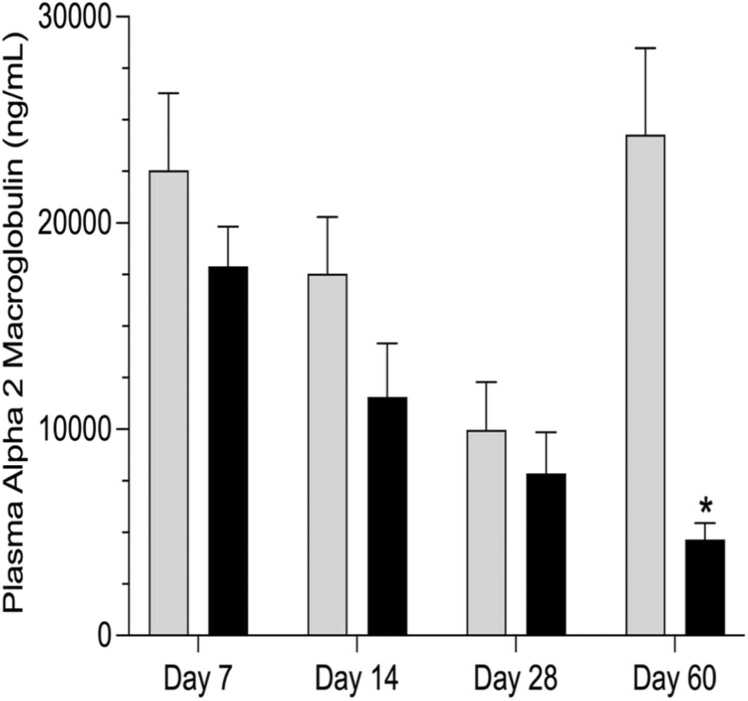
Time-course of change in systemic inflammation measured using plasma alpha 2 macroglobulin in male Sprague Dawley rats after an oral daily exposure to 0.05% v/v complex groundwater mixture (represented by black bars) or tap water (represented by gray bars) for specific time-points (7, 14, 28 and 60 days). Data are shown as mean ± standard error of the mean (n=5 rats/time/treatment). The asterisk denotes statistical significance from the control (P < 0.05; unpaired t-test, Welch correction).

Hematological indices showed a statistically significant increase in white blood cells on days 7 (5.27 ± 0.21 ×10^9^/L) and 60 (6.28 ± 0.30 ×10^9^/L), representing a 1.4-fold and 1.2-fold change, respectively (Table 3). Hematological differentials in the exposed groups showed statistically significant lymphocytosis on day 7 (4.79 ± 0.19 ×10^9^/L) and neutrophilia on day 60 (1.07 ± 0.13 ×10^9^/L) when compared to their controls, which denote a 1.5-fold and 1.9-fold change, respectively. No statistically significant changes were observed in the red blood cell indices at various time points (Table 3). In contrast, plasma alpha 2 macroglobulin (A2M) was statistically significantly reduced (5.2-fold change, p<0.05) in the exposed group relative to the control group on day 60 (Fig. 1). Conversely, there were no statistically significant changes in plasma A2M between the exposed and control group on days 7, 14, and 28 (Fig. 1). Histologically, no lesions in the liver were observed (data not shown).

**Table 3:** Time-course of 0.05% (v/v) contaminated groundwater in drinking water effects on hematological indices in male Sprague Dawley rats.

**Parameters**	**Day 7**		**Day 14**		**Day 28**		**Day 60**	
	**Control**	**Exposed**	**Control**	**Exposed**	**Control**	**Exposed**	**Control**	**Exposed**
RBC (x10^12^/L)	3.68 ± 0.17	4.01 ± 0.23	4.63 ± 0.31	4.29 ± 0.37	5.07 ± 0.31	4.96 ± 0.21	5.71 ± 0.34	5.33 ± 0.15
WBC (x10^9^/L)	3.87 ± 0.51	5.27 ± 0.21*	5.31 ± 0.72	3.87± 1.06	5.82 ± 0.97	8.50 ± 1.37	5.08 ± 0.15	6.28 ± 0.30*
*WBC Differentials (x10*^*9*^*/L)*								
Neutrophils	0.56 ± 0.06	0.29 ± 0.16	0.95 ± 0.18	0.52 ± 0.15	0.69 ± 0.14	1.43 ± 0.38	0.55 ± 0.06	1.07 ± 0.13*
Lymphocytes	3.17 ± 0.41	4.79 ± 0.19*	4.28 ± 0.66	3.35 ± 0.78	5.01 ± 0.85	6.89 ± 1.22	4.36 ± 0.18	4.92 ± 0.46

Data are presented as mean ± SE (n= 5 rats/group). Asterisk denotes statistical significance from control (P < 0.05; unpaired t-test, Welch correction). WBC = white blood cells; RBC = red blood cells.

### 3.5 Time-course experiment: effect of the complex mixture on oxidative stress in tissues

**Fig. 2:**
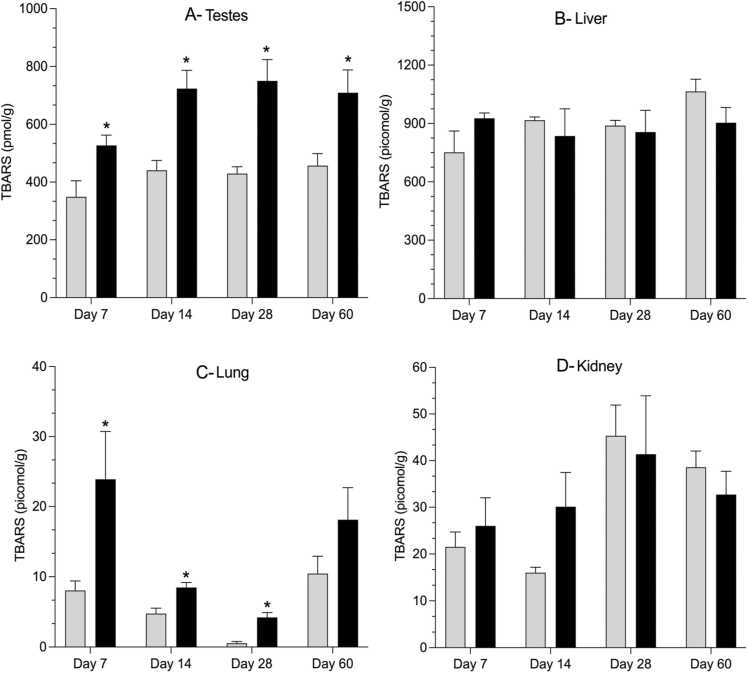
Time-course of change in tissue lipid peroxidation determined using thiobarbituric acid reactive substances (TBARS) assay in the testes (A), liver (B), lung (C) and kidney (D) in male Sprague Dawley rats after an oral daily exposure to 0.05% v/v complex groundwater mixture (represented by black bars) or tap water (represented by gray bars) for specific time-points (7, 14, 28 and 60 days). Data are shown as mean ± standard error of the mean (n=5 rats/time/treatment). The asterisk denotes statistical significance from the control (P < 0.05; unpaired t-test, Welch correction).

Testicular tissues assessed on days 7, 14, 28, and 60 showed a statistically significant elevation of MDA (1.5-fold, 1.6-fold, 1.7-fold and 1.6-fold change, respectively) in the exposed groups compared to the controls (Fig. 2). Similarly, the lungs showed a statistically significant increase in MDA on days 7, 14, and 28 (3-fold, 1.8-fold, and 7.9-fold change, respectively) in the exposed group compared to the control. However, no statistically significant differences in MDA levels were observed in the kidneys or the liver (Fig. 2).

### 3.6 Time-course experiment: effect of the complex mixture on glomerular function

**Fig. 3:**
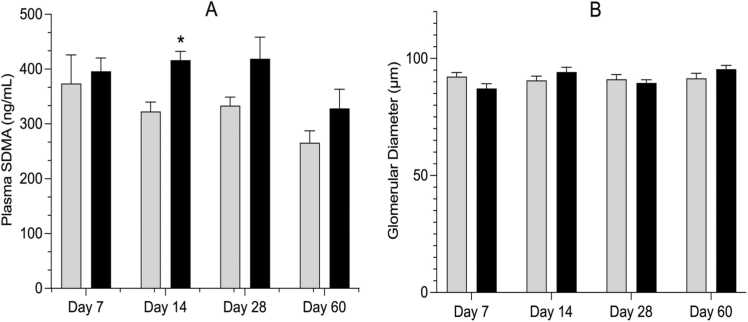
Time-course of change in glomerular toxicity determined using the plasma symmetric dimethylarginine (SDMA) in panel A and glomerular diameter (panel B) in male Sprague Dawley rats after an oral daily exposure to 0.05% v/v complex groundwater mixture (represented by black bars) or tap water (represented by gray bars) for specific time-points (7, 14, 28 and 60 days). Data are shown as mean ± standard error of the mean (n=5 rats/time/treatment). The asterisk denotes statistical significance from control (P < 0.05; unpaired t-test, Welch correction).

On day 14, the concentration of plasma symmetric dimethylarginine, which serves as an indicator of glomerulotoxicity, showed a statistically significant 1.3-fold increase in the exposed group (Fig. 3). The complex mixture did not increase plasma symmetric dimethyl arginine (SDMA) levels in a time-dependent manner, as SDMA was not elevated at any of the other time points in this study. Similarly, there were no statistically significant differences in the glomerular diameter (Fig. 3). Moreover, there were no differences among treatment groups at any time point in other kidney histological parameters, such as tubular diameter and interstitial spaces, nor any evidence of increased histopathology (data not shown).

### 3.7 Time-course experiment: effect of the complex mixture on gonadotoxicity

**Fig. 4:**
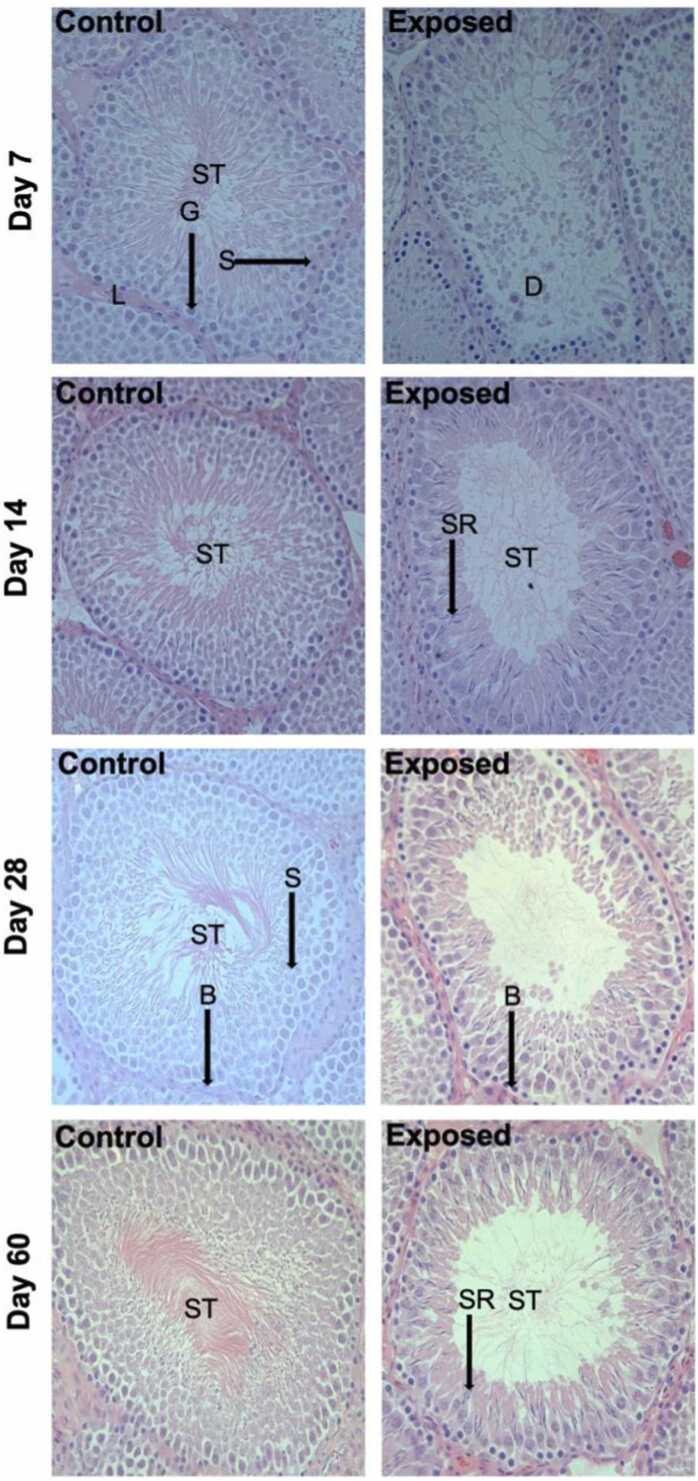
Representative micrographs of the time-course of change in testicular histopathology in male Sprague Dawley rats after an oral daily exposure to 0.05% v/v complex groundwater mixture (exposed) or tap water (control) for specific time-points (7, 14, 28 and 60 days). Testicular sections were 4 μm thick and stained with hematoxylin and eosin. Arrows indicate various histological regions and cells in the testes; spermatid retraction (SR), seminiferous tubule (ST), Sertoli cells (S), germ cells (G), basal cells (B), Leydig cells of interstitium (L).

**Fig. 5:**
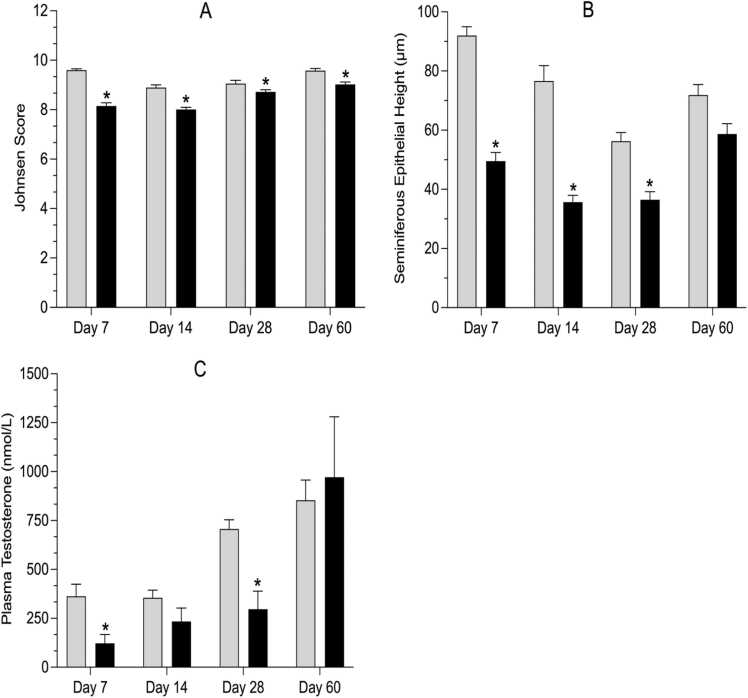
Time-course of change in testicular toxicity determined through Johnsen scores (panel A), the seminiferous epithelial height (panel B) and plasma testosterone concentrations (panel C) in male Sprague Dawley rats after an oral daily exposure to 0.05% v/v complex groundwater mixture (represented by black bars) or tap water (represented by gray bars) for specific time-points (7, 14, 28 and 60 days). Data are shown as mean ± standard error of the mean (n=5 rats/time/treatment). The asterisk denotes statistical significance from the control (P < 0.05; unpaired t-test, Welch correction).

Histological assessment of testicular maturation and pathology revealed significant effects of treatment with contaminated groundwater that became progressively more severe with increasing exposure time (Fig. 4). On day 7, the exposed group showed a distorted architecture of cells involved in spermatogenesis. The exposed groups at all time points showed reduced mature spermatocytes in the seminiferous tubules and a decreased number of cells involved in spermatogenesis. Days 14 and 60 showed evidence of retracted and nonprogressive spermatids during spermatogenesis. On day 7, the exposed rats showed evidence of degeneration of seminiferous tubules. Reactive hyperplasia of the basal cells or spermatogonia was observed in the exposed groups at all time points. Quantitative assessment of sperm maturation using Johnsen scores revealed that maturation within testicular seminiferous tubules was significantly impaired at all time points, particularly at days 7 and 14 (0.9–1-fold decrease; Fig. 5). Specifically, the functional epithelial height within the seminiferous tubules was significantly reduced in the exposed groups on days 7, 14, and 28 (0.5-fold, 0.4-fold, and 0.7-fold changes, respectively; Fig. 5). Moreover, plasma testosterone concentrations were significantly decreased in the exposed rats compared to the control on days 14 and 28 (0.3-fold and 0.4-fold changes, respectively; Fig. 5). It should be emphasized that this testicular histopathology occurred in rats orally exposed to a complex groundwater mixture that did not show any significant changes in testicular weight (Table 2), and no change in the gross appearance of the testes between the exposed and control groups was noted.

## 4 Discussion

The most important findings of this study were time-dependent testicular toxicity and time-independent effects on nephrotoxicity, immunotoxicity, and pulmonary toxicity after oral exposure to contaminated groundwater from the study site. The dose of complex groundwater used in this time-course study was determined in a range-finder study that identified the most sensitive target organ effect, cardiotoxicity, at a sub-lethal dose of 0.05% v/v. In a subsequent time-course study, we examined the adverse effects on target organs following oral exposure to 0.05% v/v of a complex groundwater mixture from a pesticide formulation and storage site in male rats. There was consistency in the full chemical characterization of contaminant mixtures from this study (Table 1) with those performed previously in 2019 as well as in 2021, 6 months after the current study’s water collection and analyses [11]. While this consistency gives confidence to predict toxicity from the detected parent compounds, we acknowledge potential effects from unknown contaminants such as breakdown products of parent toxicants that might contribute to groundwater mixture toxicity. The complex groundwater mixture did not cause any obvious toxicity based on weekly body weight, urine output, feed, and water intake in rats, and thus gave little indication of gross toxicity in both the range-finder and main time-course studies. However, in the time-course study, histological and/or biochemical indicators of toxicity were observed primarily in the testes, but with minor increases in the white blood cell count as well as lipid peroxidation in some target organs.

### 4.1 Gonadal toxicity

The reduced seminiferous epithelial height detected in this study is an important histomorphometric indicator of damage during spermatogenesis [19], [23]. The reduced Johnsen scores observed in the groups exposed to 0.05% v/v contaminated groundwater have been closely linked to the degeneration of spermatids, spermatogonia and spermatozoa [1], [24], [30], confirming impaired spermatogenesis. There was also significantly elevated malondialdehyde (MDA) within the testes at all time-points. Compared to previous studies that orally exposed rats to single contaminants involving 2,4-D [14], [8] and petroleum F2 fractions [10], oxidative stress in testicular tissues was identified as the mechanism of toxicity. In these studies, the concentrations of 2,4-D (12 mg/kg and 130 mg/kg) and hydrocarbon F2 fractions (800 mg/kg) were substantially higher than those found in this study’s complex groundwater mixture even before dilution. Thus, the testicular toxicity observed in this study might instead be due to complex interactions among the contaminants within the mixture or driven by unknown contaminants or active breakdown products in the groundwater.

Further biochemical assessment following testicular histopathology showed significantly reduced plasma testosterone levels on days 7 and 28 compared with the controls. The reduction in plasma testosterone could be attributed to the significant increase in testicular MDA resulting from oxidative stress in steroidogenic pathways in Leydig cells and supporting cells [14], [8]. Suppression of testosterone production has been shown to correlate strongly with reduced antioxidants, since Leydig cells harbor the majority of the testicular antioxidant systems [10], [18]. Therefore, it is possible that the destruction of Leydig cells leads to a reduction in testosterone production and antioxidant systems, predisposing the testes to further oxidative damage [25]. In our study, no significant reduction in testicular weight was observed at any time point in the exposed rats. However, multiple studies have identified a significant reduction in testicular weight following oral exposure to contaminants, including high concentrations of 2,4-D at a dietary intake of 300 mg/kg [7], 100 mg/kg, and 200 mg/kg [22]. Taken together, the individual concentrations of the 2,4-D and petrochemical F2 fractions in the groundwater mixture were significantly lower than those reported in previous studies. Hence, the drivers of testicular toxicity are most likely unknown parent compounds and metabolites or interactions among compounds. The authors further acknowledge the limitation of not including female rats, which might have detected comparable gonadal toxicity outcomes.

### 4.2 Immune & hepatic toxicity

The acute inflammatory phase protein, alpha 2 macroglobulin, in the group that was exposed to 0.05% groundwater mixture, showed a significant reduction of 5.2-fold when compared to the control group on day 60. An explanation for the repression of alpha 2 macroglobulin could be the inhibition of cytokines such as interleukins 6 and 1, which are responsible for the induction of acute phase proteins [12], [32]. If reduced cytokines are an explanation for reduced alpha 2 macroglobulin, this should be coincident with generalized immunosuppression. However, there was no change in spleen weight or in spleen histopathology (data not shown). Hepatopathy has been shown to cause a reduction in plasma alpha 2 macroglobulin [17], [20]. However, in this study, there was no evidence of hepatic impairment and no hepatic histopathology; hence, hepatopathy could not be directly linked to the reduction in plasma alpha 2 macroglobulin concentration. Similarly, a previous study from our group using extracts of contaminated soil from this same study site showed no change in direct or indirect bilirubin (Boamah et al., 2024). Moreover, mild lymphocytosis and neutrophilia were observed in the current study, arguing against immunosuppression and instead indicating impaired hepatic synthetic capacity, which should be examined in future studies.

### 4.3 Renal & pulmonary toxicity

Plasma symmetric dimethylarginine (SDMA) levels were significantly elevated in the groups exposed to 0.05% groundwater mixture on day 14. Plasma SDMA correlates strongly with the glomerular filtration rate (GFR), and an increase in SDMA is indicative of a compromised GFR [13]. On the contrary, no histopathological changes were observed in the renal tubules, glomeruli and interstitium. This discrepancy could be ascribed to the dose of the groundwater mixture, which resulted in functional but not structural nephrotoxicity. Contaminants identified in groundwater including petrochemical F2 fractions and 2,4-D have been shown to induce nephrotoxicity in rats [2], [27]. However, once again, the relatively low concentration in the groundwater mixture are unlikely drivers of the nephrotoxicity. Hence other unknown constituents in the mixture maybe responsible.

Our study observed mild pulmonary cytotoxicity in the exposed groups following oral exposure to the complex groundwater mixture. The pulmonary cytotoxicity was evident by a significant increase in MDA concentration with a decreasing trend from day 7–28 in comparison to the control. The decreasing in MDA concentration could be due to the time-dependent recovery of pulmonary cells following oxidative stress [29].

### 4.4 Potential drivers of groundwater toxicity & comparison to soil toxicity

The pattern of toxicity we found in the time-course experiments using contaminated groundwater in the drinking water for rats did not produce the same toxicity we observed previously using an extract of contaminated soil from this site [4]. The disparity in adverse effects between both studies could be ascribed to the differences in exposure duration and concentrations in exposed soil extracts and groundwater since similar contaminants were identified in both media. However, unknown compounds and multiple transformed products might contribute to the target organ toxicities identified in both studies. The nephrotoxicity, immunotoxicity, and testicular toxicities observed in the current time-course experiment are consistent with 2,4-D and petrochemical F2 fraction toxicity, but as discussed above, the concentrations of these chemicals were below the levels needed for toxicity.

## 5 Conclusion

Only nephrotoxicity was observed in both the current study that used contaminated groundwater and in our previous study using soil extracts from the same site in male Sprague Dawley rats. Thus, only the observation of nephrotoxicity supports our original hypothesis that oral exposure to 0.05% v/v complex groundwater mixture will induce similar toxicity. The major target organ for the groundwater was the testis, but it is unknown if the soil extracts would have exerted similar toxicity since gonads were not examined in the previous study. In the current study, a range-finder study identified a sub-lethal dose of 0.05% complex groundwater mixture as the safest for the main time-course study since the lowest dose showed cardiotoxicity. In the main time-course study, the contaminated groundwater used as drinking water (0.05% v/v) showed some mild nephrotoxicity, immune system effects and pulmonary toxicity in addition to the larger testicular toxicity. Time-dependent impacts were observed in the effects of oxidative stress on the testes and pulmonary toxicity. In the testicular assessment, days 7, 14, and 28 were critical time-points of toxicities. Moreover, in this study, the adverse outcomes that were observed reveal a major limitation in the use of compound-by-compound risk assessment approach, which would have predicted *no* adverse effects based on the concentrations of the individual compounds that were identified. This limitation highlights the benefits of instead using effects-directed and non-target analyses in assessing toxicity of complex contaminated sites. Further development of integrated approaches including animal (in-vivo) models in scenarios of complex mixtures with the potential for degradation products or other unknowns to be present would increase accuracy and relevance of the human health risk assessment process.

## CRediT authorship contribution statement

**Markus Hecker:** Writing – review & editing, Funding acquisition. **Mark Hanson:** Writing – review & editing, Funding acquisition. **Patrick Campbell:** Writing – review & editing. **Rachel Peters:** Writing – review & editing. **Ahmad N. Al-Dissi:** Writing – review & editing, Formal analysis. **Lynn Weber:** Writing – review & editing, Supervision, Resources, Project administration, Methodology, Investigation, Conceptualization. **Bright Boamah:** Writing – review & editing, Writing – original draft, Visualization, Validation, Methodology, Investigation, Formal analysis, Data curation, Conceptualization. **Steven Siciliano:** Resources, Project administration, Funding acquisition, Conceptualization. **Natacha Hogan:** Writing – review & editing, Funding acquisition.

## Author declaration

The work contained within this manuscript has not submitted for publication in any other journal or publication.

## Declaration of Generative AI and AI-assisted technologies in the writing process

The authors declare that no AI was used in any writing of this manuscript.

## Declaration of Competing Interest

The authors declare the following financial interests/personal relationships which may be considered as potential competing interests: Steve Siciliano reports financial support was provided by Federated Cooperatives Ltd. Rachel Peters reports a relationship with Federated Cooperatives Ltd that includes: employment. If there are other authors, they declare that they have no known competing financial interests or personal relationships that could have appeared to influence the work reported in this paper.

## Data Availability

Data will be made available on request.
