# Supplementary material for: Time-course of oral toxicity to contaminated groundwater in male Sprague Dawley rats
Source: Toxicol Rep. 2024 May 17;12:584–93. doi: 10.1016/j.toxrep.2024.05.002 (PMC11134541; doi:10.1016/j.toxrep.2024.05.002)
Supplement: Supplementary file 1 — Supplementary material [file mmc1.docx]

**SUPPLEMENTAL MATERIALS**

**Time-Course of Oral Toxicity to Contaminated Groundwater in Male Sprague Dawley Rats**

**B. Boamah, S. Siciliano, N. Hogan, M. Hecker,**

**M. Hanson, P. Campbell, R. Peters, A.N. Al-Dissi and L. Weber**

**Study site with sites of test sample collection indicated**

The study site was an industrial site with historical and current contamination as indicated below. Contaminated groundwater samples for the current study were taken from a well at TH16-02 indicated below in Figure S1.

**

**

Figure S1 The study site was a pesticide formulation and storage site, located within an urban area in Canada (border of the property indicated by blue demarcation). The complex groundwater mixture of contaminants identified included pesticides, hydrocarbons, and heavy metals. This study evaluated 0.05% v/v of groundwater collected from a highly impacted zone on site (indicated on the map as TH16-02). Sites A and B denote areas in a previous study by *Boamah et al.,2023* where soil extracts were collected. Areas S and H denote areas of storage tanks for raw materials and holding tanks for unfinished products respectively.

**Table S1** Complete chemical analyses of organic compounds in groundwater mixture collected from the high impact well at the industrial site in Fall 2020.

| **Herbicides** | **Methodology** | **Limit of Detection (LOD)** | **Units** | **High Impact Well (Th16-02)** |
| --- | --- | --- | --- | --- |
| Clopyralid |  | 0.0010 | mg/L | 0.0181 |
| Dicamba |  | 0.0050 | mg/L | 0.194 |
| Mecoprop |  | 0.10 | mg/L | 2.52 |
| MCPA |  | 0.0010 | mg/L | 0.0017 |
| 2,4-D |  | 0.0010 | mg/L | 0.0070 |
| Bromoxynil | Gas chromatography/mass spectrometry | 0.050 | mg/L | 0.161 |
| Triclopyr |  | 0.0010 | mg/L | <LOD |
| 2,4,5-T |  | 0.0010 | mg/L | 0.0060 |
| 2,4,5-TP |  | 0.0010 | mg/L | 0.0093 |
| Picloram |  | 0.0010 | mg/L | 0.0783 |
| 2,4-DB |  | 0.10 | mg/L | 1.58 |
| 2,4-DP |  | 0.050 | mg/L | 0.703 |
| Dinoseb |  | 0.0010 | mg/L | <LOD |
| MCPB |  | 0.0010 | mg/L | 0.0052 |
| **Pesticides (Water)** |  |  |  |  |
| Trifluralin | Gas chromatography/mass spectrometry | 0.00010 | mg/L | <LOD |
| Triallate |  | 0.00020 | mg/L | <LOD |
| Fluazifop-p-butyl |  | 0.00010 | mg/L | <LOD |
| Diclofob-methyl |  | 0.00010 | mg/L | <LOD |
| Ethalfluralin |  | 0.00010 | mg/L | <LOD |
| **Volatile Organic Compounds (Water)** |  |  |  |  |
| Acetone |  | 0.050 | mg/L | 0.194 |
| Benzene |  | 0.00050 | mg/L | 0.0111 |
| Bromobenzene |  | 0.0010 | mg/L | <LOD |
| Bromochloromethane |  | 0.0010 | mg/L | <LOD |
| Bromodichloromethane | Gas chromatography/mass spectrometry | 0.00050 | mg/L | <LOD |
| Bromoform |  | 0.0010 | mg/L | <LOD |
| Bromethane |  | 0.0010 | mg/L | <LOD |
| n-Butylbenzene |  | 0.0010 | mg/L | 0.0085 |
| Sec-Butylbenzene |  | 0.0010 | mg/L | <LOD |
| Tert-Butylbenzene |  | 0.0010 | mg/L | <LOD |
| Carbon disulfide |  | 0.0050 | mg/L | <LOD |
| Chlorobenzene |  | 0.00050 | mg/L | <LOD |
| Chloroethane |  | 0.0030 | mg/L | <LOD |
| Chloroform |  | 0.0010 | mg/L | <LOD |
|  |  |  |  | (Continued) |
| **Volatile Organic Compounds (Water)** | **Methodology** | **Limit of Detection (LOD)** | **Units** | **High Impact Well (Th16-02)** |
| Chloromethane |  | 0.00050 | mg/L | <LOD |
| 2-Chlorotoluene |  | 0.0050 | mg/L | <LOD |
| 4-Chlorotoluene |  | 0.020 | mg/L | <LOD |
| Dibromochloromethane |  | 0.0010 | mg/L | <LOD |
| 1,2-Dibromo-3-chloropropane |  | 0.00050 | mg/L | <LOD |
| 1,2-Dibromoethane |  | 0.0010 | mg/L | <LOD |
| Dibromomethane |  | 0.0010 | mg/L | <LOD |
| 1,2-Dichlorobenzene | Gas chromatography/mass spectrometry | 0.0010 | mg/L | <LOD |
| 1,3-Dichlorobenzene |  | 0.00050 | mg/L | <LOD |
| 1,4-Dichlorobenzene |  | 0.0010 | mg/L | <LOD |
| Dichlorodifluoromethane |  | 0.0010 | mg/L | <LOD |
| 1,1-Dichloroethane |  | 0.0010 | mg/L | <LOD |
| 1,2-Dichloroethane |  | 0.00050 | mg/L | <LOD |
| 1,1-Dichloroethene |  | 0.00050 | mg/L | <LOD |
| Cis-1,2-Dichloroethene |  | 0.00050 | mg/L | <LOD |
| Trans-1,2-Dichloroethene |  | 0.0010 | mg/L | <LOD |
| Dichloromethane |  | 0.0010 | mg/L | <LOD |
| 1,2-Dichloropropane |  | 0.0050 | mg/L | 0.0120 |
| 1,3-Dichloropropane |  | 0.0010 | mg/L | <LOD |
| 2,2-Dichloropropane |  | 0.0010 | mg/L | <LOD |
| 1,1-Dichloropropane |  | 0.0010 | mg/L | <LOD |
| Cis-1,3-Dichloropropene |  | 0.0010 | mg/L | <LOD |
| Trans-1,3-Dichloropropene |  | 0.0010 | mg/L | <LOD |
| Ethylbenezene |  | 0.0010 | mg/L | <LOD |
| F1 |  | 0.00050 | mg/L | 0.0172 |
| Hexachlorobutadiene |  | 0.10 | mg/L | 0.36 |
| Hexane |  | 0.0010 | mg/L | <LOD |
| 2-Hexanone |  | 0.020 | mg/L | <LOD |
| Isopropylbenzene |  | 0.0010 | mg/L | 0.0018 |
| 4-Isopropyltoluene |  | 0.0010 | mg/L | 0.0019 |
| MEK |  | 0.020 | mg/L | 0.036 |
| MIBK |  | 0.020 | mg/L | <LOD |
| Styrene |  | 0.00050 | mg/L | <LOD |
| 1,1,1,2-Tetrachloroethane |  | 0.0010 | mg/L | <LOD |
| 1,1,2,2-Tetrachloroethane |  | 0.00050 | mg/L | <LOD |
|  |  |  |  | (Continued) |
| **Volatile Organic Compounds (Water)** | **Methodology** | **Limit of Detection (LOD)** | **Units** | **High Impact Well (Th16-02)** |
| Tetrachloroethene |  | 0.00050 | mg/L | <LOD |
| Toluene |  | 0.00050 | mg/L | 0.00074 |
| 1,2,3-Trichlorobenzene |  | 0.0010 | mg/L | <LOD |
| 1,2,4-Trichlorobenzene | Gas chromatography/mass spectrometry | 0.0010 | mg/L | <LOD |
| 1,1,1-Trichloroethane |  | 0.00050 | mg/L | <LOD |
| 1,1,2-Trichloroethane |  | 0.00050 | mg/L | <LOD |
| Trichloroethene |  | 0.00050 | mg/L | <LOD |
| Trichlorofluoromethane |  | 0.0010 | mg/L | <LOD |
| 1,2,3-Trichloropropane |  | 0.0010 | mg/L | <LOD |
| 1,2,4-Trimethylbenzene |  | 0.0010 | mg/L | 0.105 |
| 1,3,5-Trimethylbenzene |  | 0.0010 | mg/L | 0.0339 |
| Vinyl Chloride |  | 0.00050 | mg/L | <LOD |
| M+P-Xylenes |  | 0.00040 | mg/L | 0.0210 |
| o-Xylene |  | 0.00050 | mg/L | 0.0185 |
| **Hydrocarbons (Water)** |  |  |  |  |
| F2 (C10 - C16) |  | 0.10 | mg/L | 11.0 |
| F3 (C16 - C34) |  | 0.25 | mg/L | 2.15 |
| F4(C34 - C50) |  | 0.25 | mg/L | <LOD |
|  |  |  |  |  |

**Table S2** Chemical analyses of heavy metals screened in groundwater mixture collected from the high impact well at the industrial site in Fall 2020.

| **Dissolved Metals (Water)** | **Methodology** | **Limit of Detection (LOD)** | **Units** | **Concentration in High Impact Well (Th16-02)** |
| --- | --- | --- | --- | --- |
| Aluminium |  | 0.0010 | mg/L | 0.0025 |
| Antimony |  | 0.00010 | mg/L | 0.00051 |
| Arsenic |  | 0.00010 | mg/L | 0.00402 |
| Barium |  | 0.00010 | mg/L | 0.0345 |
| Beryllium |  | 0.00010 | mg/L | <LOD |
| Bismuth |  | 0.000050 | mg/L | <LOD |
| Boron |  | 0.010 | mg/L | 0.299 |
| Cadmium |  | 0.0000050 | mg/L | 0.0000096 |
| Calcium |  | 0.050 | mg/L | 103 |
| Cesium |  | 0.000010 | mg/L | 0.000010 |
| Chromium |  | 0.00010 | mg/L | 0.00018 |
| Cobalt |  | 0.00010 | mg/L | 0.00388 |
| Copper | Collision/reaction cell (CRC) tandem inductively coupled plasma mass spectrometry (ICPMS) | 0.00020 | mg/L | 0.00684 |
| Iron |  | 0.010 | mg/L | 0.025 |
| Lead |  | 0.000050 | mg/L | 0.000209 |
| Lithium |  | 0.0010 | mg/L | 0.261 |
| Magnesium |  | 0.0010 | mg/L | 309 |
| Manganese |  | 0.00010 | mg/L | 0.242 |
| Molybdenum |  | 0.000050 | mg/L | 0.00700 |
| Nickel |  | 0.00050 | mg/L | 0.256 |
| Phosphorus |  | 0.030 | mg/L | 0.097 |
| Potassium |  | 0.050 | mg/L | 8.46 |
| Rubidium |  | 0.00020 | mg/L | 0.00210 |
| Selenium |  | 0.000050 | mg/L | 0.00190 |
| Silicon |  | 0.050 | mg/L | 7.59 |
| Silver |  | 0.000010 | mg/L | <LOD |
| Sodium |  | 0.050 | mg/L | 229 |
| Strontium |  | 0.00010 | mg/L | 1.35 |
| Sulfur |  | 0.50 | mg/L | 148 |
| Tellurium |  | 0.00020 | mg/L | <LOD |
| Thallium |  | 0.000010 | mg/L | 0.000032 |
| Thorium |  | 0.00010 | mg/L | <LOD |
| Tin |  | 0.00010 | mg/L | 0.00020 |
| Titanium |  | 0.00030 | mg/L | 0.00053 |
| Tungsten |  | 0.00010 | mg/L | <LOD |
| Uranium |  | 0.000010 | mg/L | 0.0255 |
| Vanadium |  | 0.00050 | mg/L | 0.00330 |
| Zinc |  | 0.0010 | mg/L | 0.0401 |
| Zirconium |  | 0.00020 | mg/L | 0.00516 |
|  |  |  |  |  |

**Range finder study**

No significant changes were observed in the final bodyweights and organ weights among the control and the 0.05%, 0.5%, and 5% groundwater-exposed rats at the end of the range finder study (Table S3).

Table S3 Range finder experiment effects on body and organ weights after oral exposure to high-impact groundwater (% v/v) or tap water (control) for 7 days in male Sprague Dawley rats.

| **Endpoints** | **Control** | **Low dose**  **(0.05%)** | **Medium dose**  **(0.5%)** | **High dose**  **(5%)** |
| --- | --- | --- | --- | --- |
| Bodyweight | 403 ± 12 | 400 ± 4 | 376 ±36 | 407 ± 2 |
| *Organ weight (mg per g BW* x *10^3^)* |  |  |  |  |
| Liver | 47.3 ± 1.4 | 47.7 ± 0.5 | 51.7 ± 5.4 | 46.8 ± 0.3 |
| Spleen | 1.9 ± 0.1 | 1.8 ± 0.1 | 1.9 ± 0.2 | 1.8 ± 0.1 |
| Heart | 3.3 ± 0.1 | 3.3 ± 0.1 | 3.6 ± 0.4 | 3.3 ± 0.1 |
| Kidney | 7.5 ± 0.2 | 7.6 ± 0.1 | 8.2 ± 0.9 | 7.5 ± 0.1 |
| Brain | 4.9 ± 0.2 | 4.9 ± 0.1 | 5.4 ± 0.6 | 4.9 ± 0.1 |
| Lungs | 4.3 ± 0.1 | 4.3 ± 0.1 | 4.7 ± 0.5 | 4.2 ± 0.2 |
| Testes | 8.7 ± 0.3 | 8.8 ± 0.1 | 9.5 ± 0.9 | 8.6 ± 0.1 |
| Food intake (g/rat/week) | 103 ± 8 | 96 ± 1 | 90 ± 9 | 98 ± 1 |
| Water intake (mL/rat/week) | 81 ± 2 | 80 ± 1 | 75 ± 7 | 81 ± 1 |

Data are presented as mean ± SE (n = 3–4 rats/group). No statistically significant differences were detected from the control (P > 0.05; Dunnett’s post-hoc after one-way ANOVA test).

No significant changes were observed among the biochemical parameters in blood (liver function,

renal function tests, electrolytes, lipid, and glucose) between the control and the 0.05%, 0.5%, and

5% groundwater-exposed groups at the end of the range finder study (Table S4).

Table S4 Range finder experiment effects of high impact groundwater (% v/v) or tap water (control) on changes in biochemical parameters in male Sprague Dawley rats after oral exposure for 7 days.

| **Endpoints** | **Control** | **Low dose**  **(0.05%)** | **Medium dose**  **(0.5%)** | **High dose**  **(5%)** |
| --- | --- | --- | --- | --- |
| Cholesterol (mmol/L) | 1.6 ± 0.2 | 1.5 ± 0.1 | 1.8 ± 0.2 | 1.7 ± 0.1 |
| Creatinine (μmol/L) | 27.8 ± 1.7 | 25.7 ± 0.3 | 25.3 ± 2.4 | 26.3 ± 1.7 |
| Urea (mmol/L) | 7.4 ± 0.4 | 7.3 ± 0.7 | 6.2 ± 0.5 | 6.4 ± 0.3 |
| Sodium (mmol/L) | 138.8 ± 1.1 | 141.7 ± 0.3 | 140 ± 0.6 | 138.3 ± 1.2 |
| Potassium (mmol/L) | 8.4 ± 0.8 | 6.3 ± 0.4 | 6.2 ± 0.3 | 7.9 ±1.1 |
| Chloride (mmol/L) | 98 ±0.4 | 99.3 ± 0.3 | 97.3 ± 0.7 | 97.3 ± 0.9 |
| Creatinine kinase (U/L) | 712 ± 233 | 652 ± 205 | 665 ± 268 | 962 ± 488 |
| Creatinine clearance | 3.3 ± 0.5 | 2.1 ± 0.7 | 2.6 ± 0.4 | 2.8 ± 0.2 |
| Glucose (mmol/L) | 15.2 ±0.9 | 13.3 ± 1.5 | 15.4 ±0.3 | 16.7 ±1.2 |
| Aspartate Phosphatase (U/L) | 241.5 ± 8.5 | 247.7 ± 16.9 | 285.9 ± 24.7 | 247.3 ± 10.4 |
| Alkaline Phosphatase (U/L) | 50 ± 5.1 | 47 ± 1.5 | 45 ± 4.9 | 53 ± 9.5 |
| Albumin (g/L) | 36.8 ± 1.3 | 39.3 ± 1.7 | 38.6 ± 1.8 | 38 ±1.5 |
| Globulin (g/L) | 16.8 ± 0.5 | 14 ± 2.5 | 13 ± 0.9 | 17 ± 1.2 |
| Total bilirubin (μmol/L) | 0.55 ± 0.16 | 0.53 ± 0.20 | 0.3 ±0.01 | 0.3 ± 0.01 |

Data are presented as mean ± SE (n = 3–4 rats/group). No statistically significant differences were detected from the control (P > 0.05; Dunnett’s post-hoc after one-way ANOVA test).

No significant changes in creatinine clearance were observed between the control and the groundwater-exposed groups (0.05%, 0.5%, and 5%) at the end of 7 days (Figure S2).


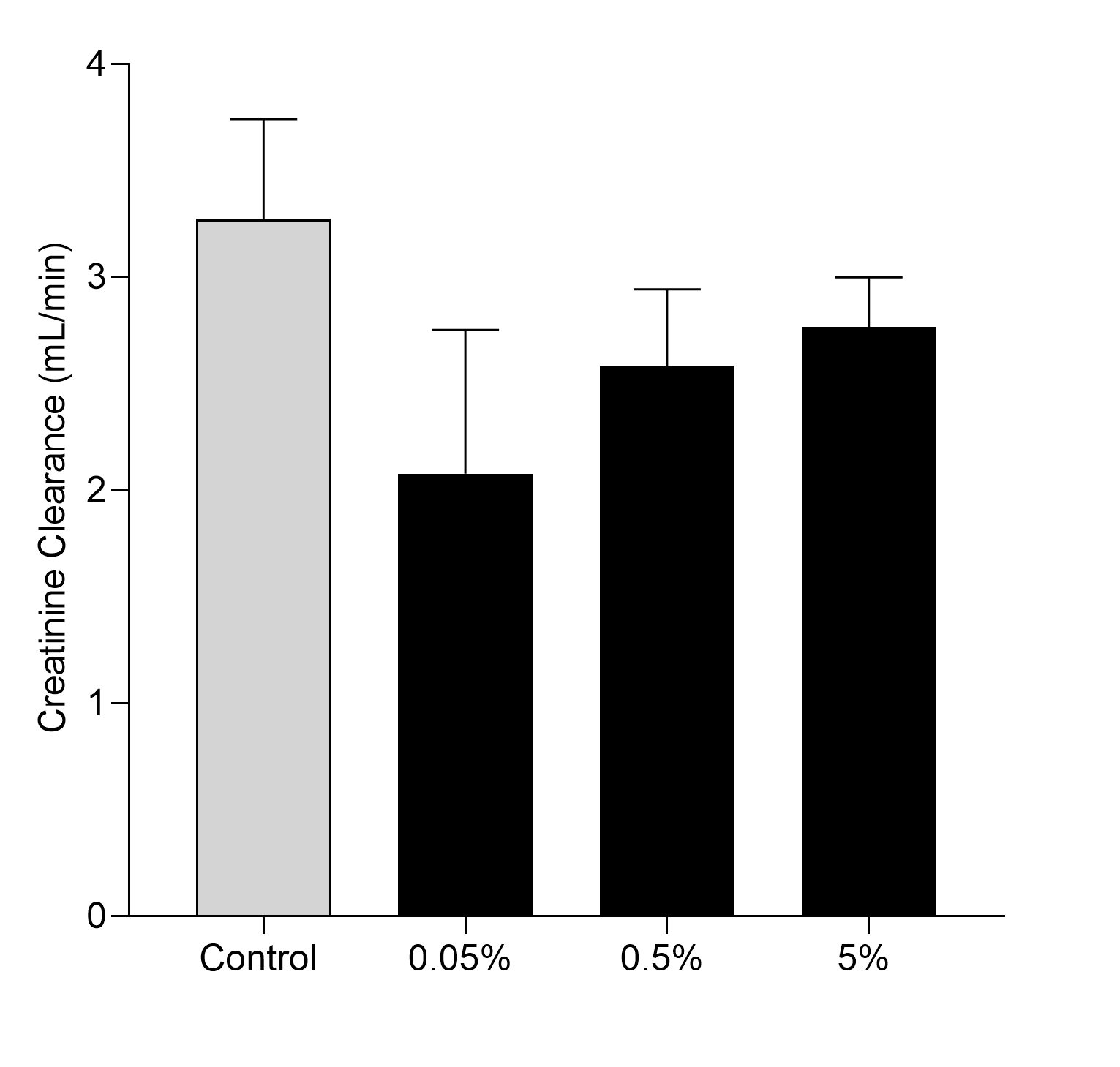


Figure S2. Range-finder experiment effects on creatinine clearance in male Sprague Dawley rats orally exposed to high impact groundwater mixture (% v/v) shown in black bars or tap water (control) shown in grey bar for 7 days. Data are shown as mean ± standard error of the mean (n=3-4/group). No statistically significant differences were detected from the control (P > 0.05; Dunnett’s post-hoc after one-way ANOVA test).

No change in QRS high was detected among groups (Figure S3A), but a significantly elevated heart rate was observed in the 0.05% and 0.5% groundwater relative to the control (Figure S3B). Similarly, a significant elevation in the PR and QTc intervals were observed following the exposure to the 0.5% and 0.05% groundwater, respectively, when compared to the control (Figure S3C and S3D).


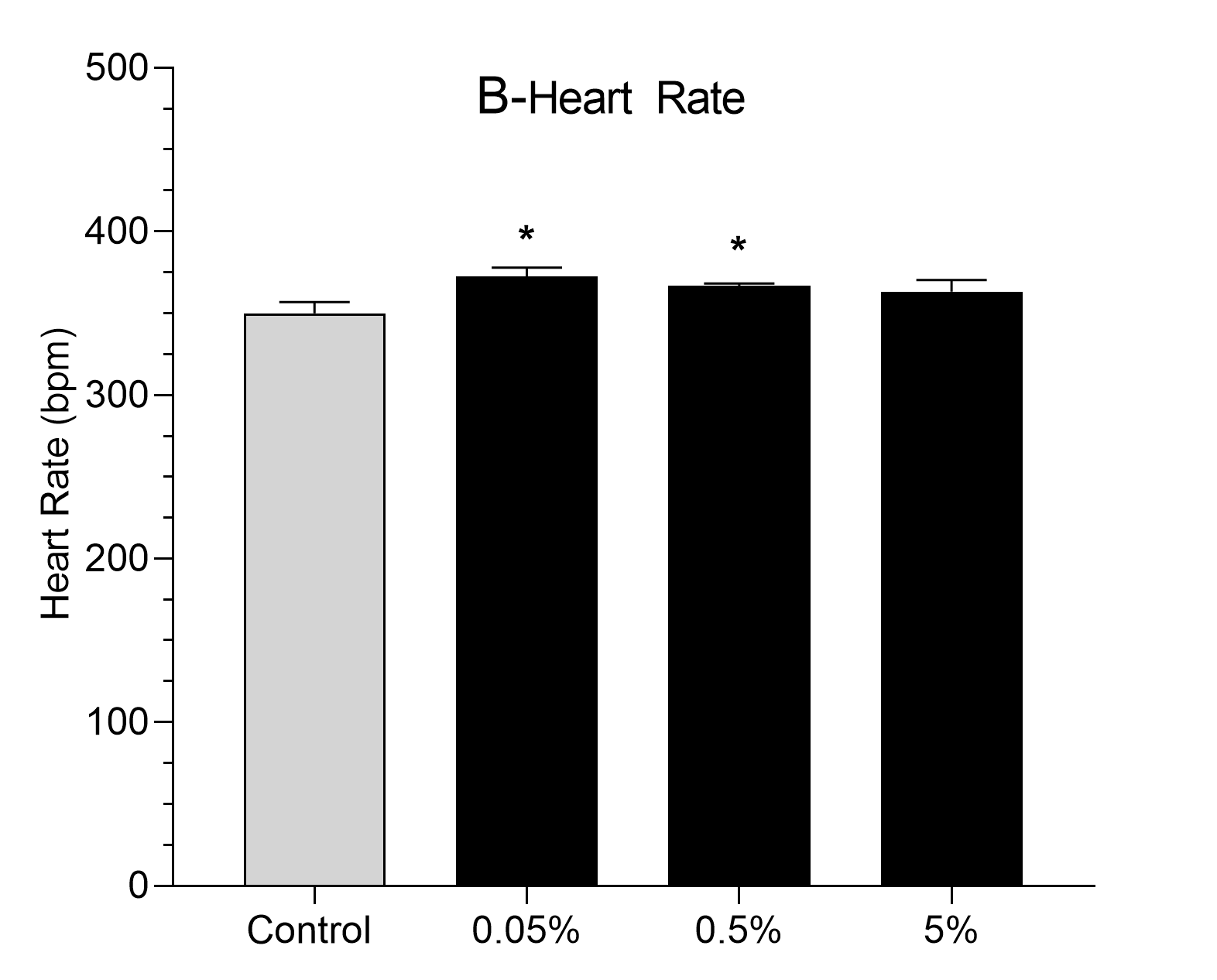

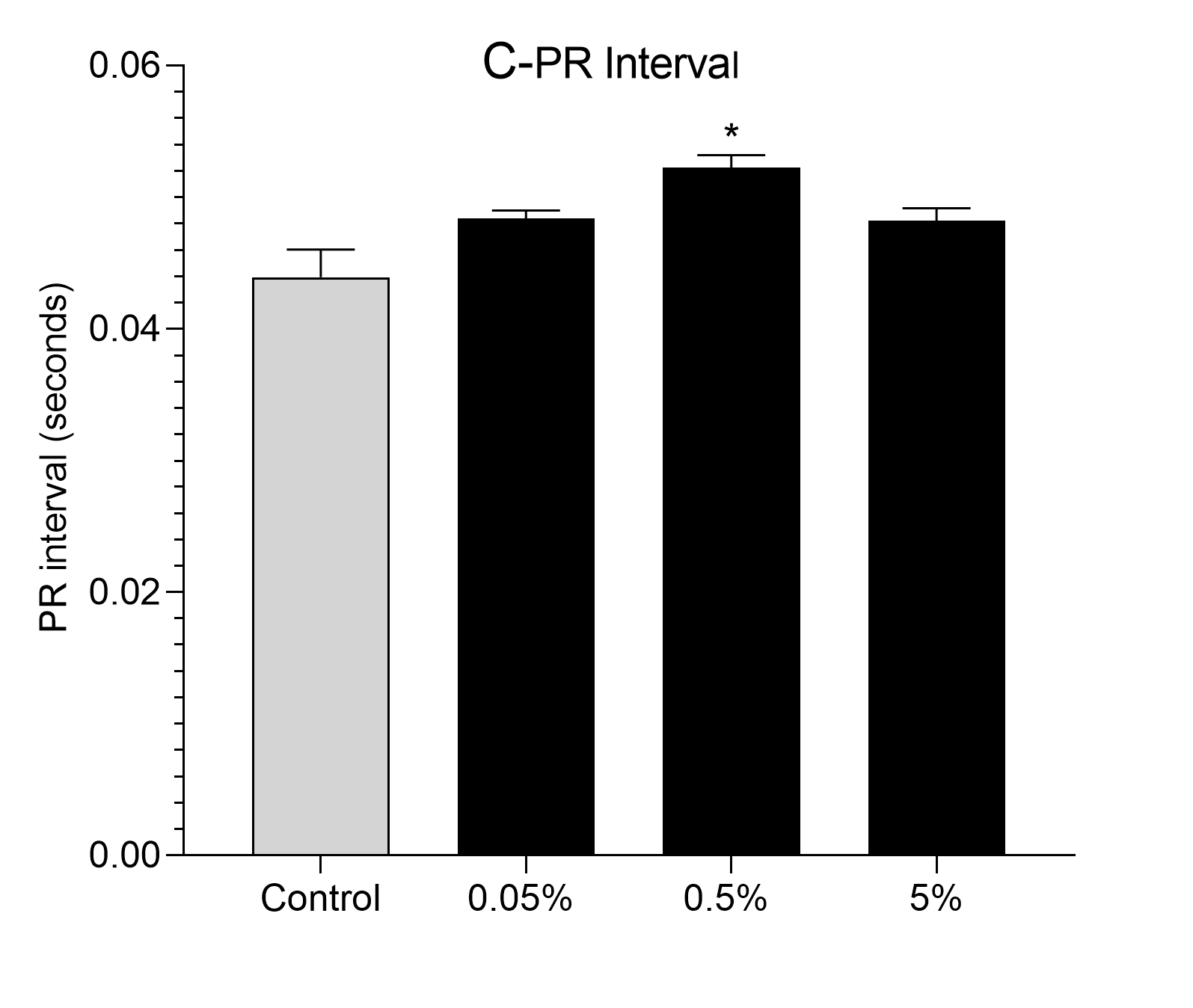

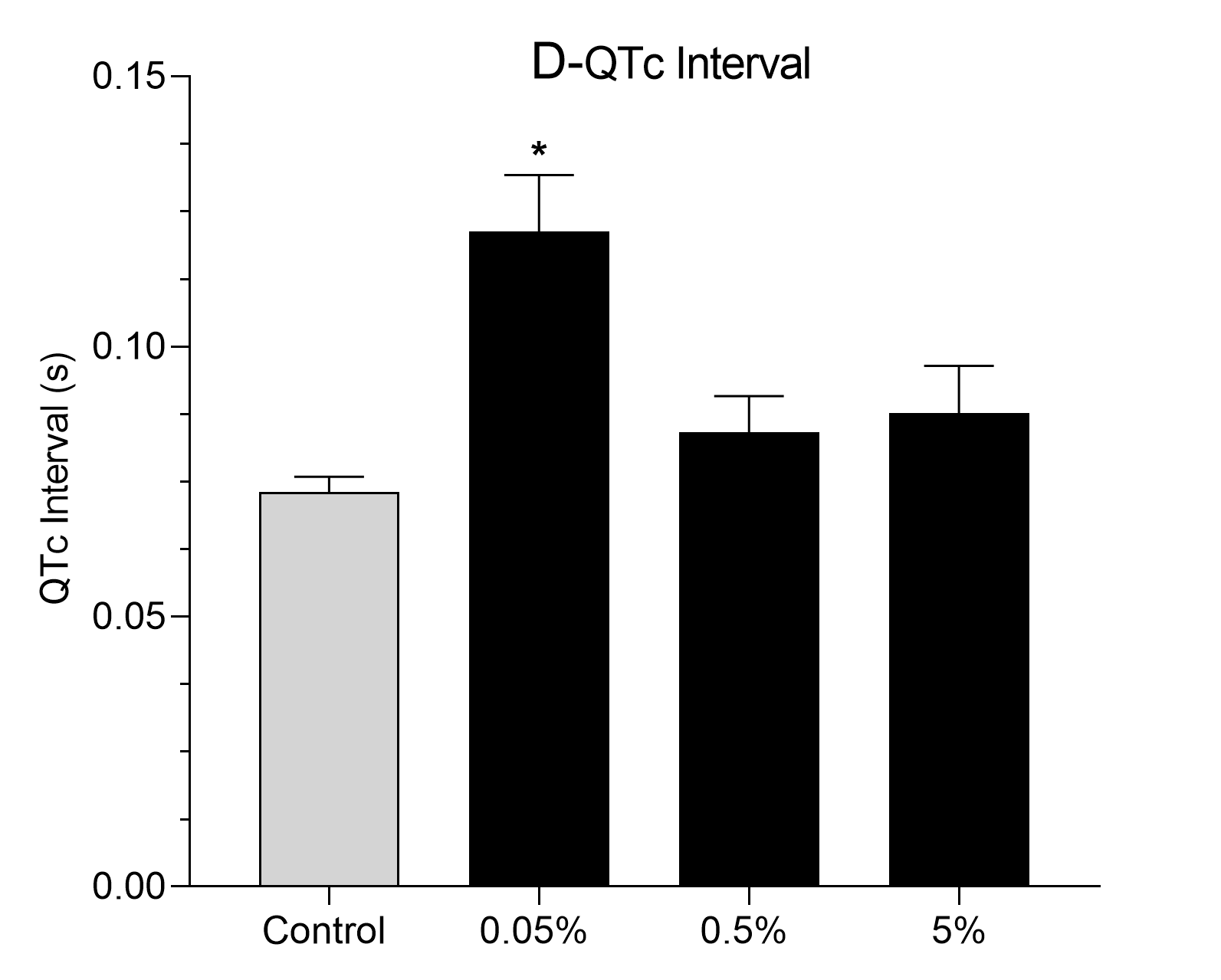

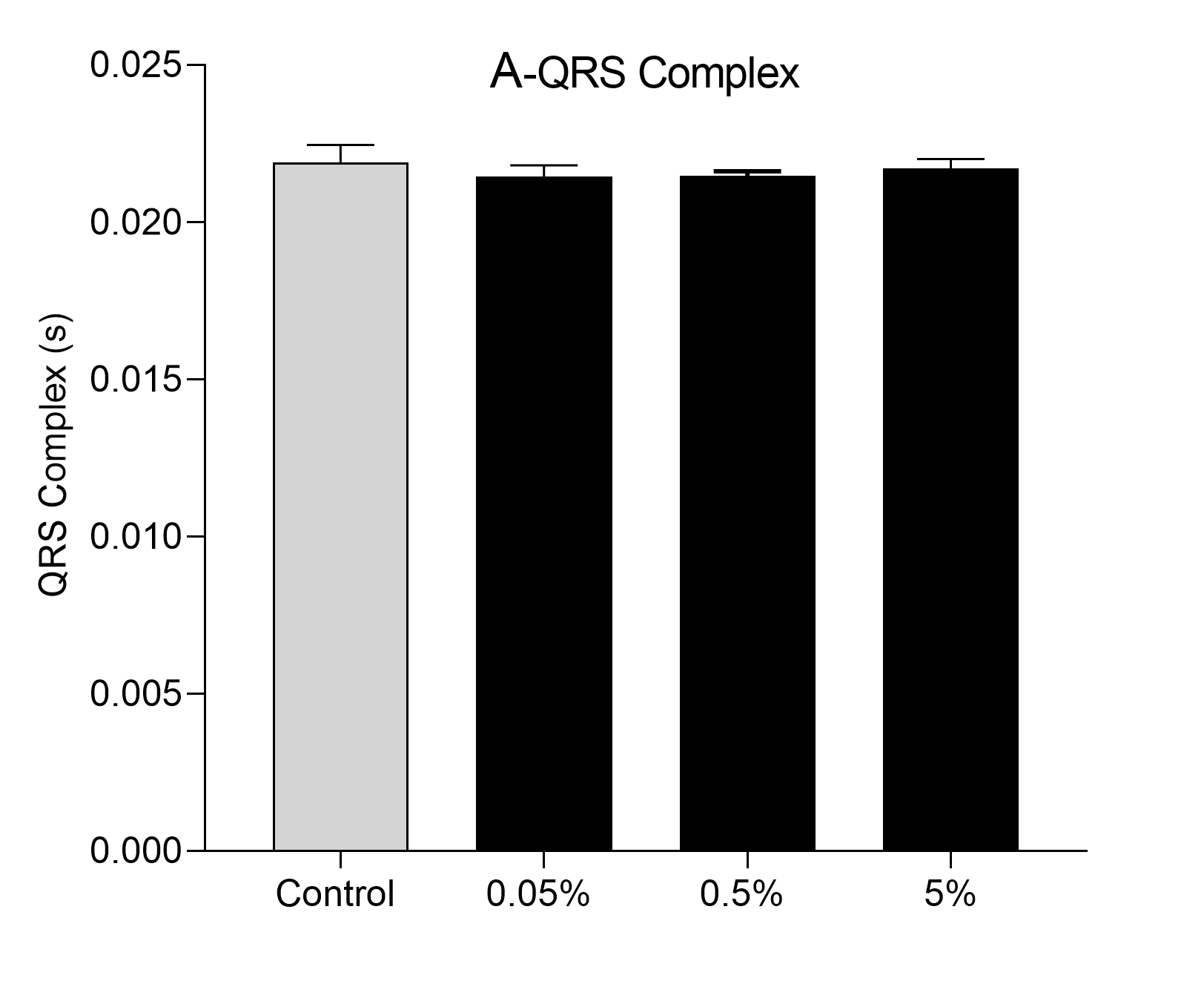


Figure S3. Range-finder experiment effects on cardiac activity determined using electrocardiogram interval analyses (A: QRS complex; B: Heart rate; C: PR interval; and D: QTc) in male Sprague Dawley rats orally exposed to high impact groundwater mixture (% v/v) represented by black bars or tap water (control) represented by grey bars for 7 days. Data are shown as mean ± standard error of the mean (n=3-4/group). The asterisk represents significant differences from the control (P < 0.05; Dunnett’s post-hoc after one-way ANOVA test).

**Time-course study**

No significant changes in feed, and water intake over the specific time-points between the control and 0.05% groundwater-exposed rats (Table S3). Similarly, no significant changes in the urine output were observed between the control and 0.05% groundwater-exposed rats over the specific time-points (Table S5).

Table S5 Time-course of 0.05% (v/v) contaminated groundwater in drinking water effects on

feed and water intake plus urine output in male Sprague Dawley rats.

| **Parameters** | **Day 7** | **Day 14** | **Day 28** | **Day 60** |
| --- | --- | --- | --- | --- |
|  | **Control Exposed** | **Control Exposed** | **Control Exposed** | **Control Exposed** |
| Feed Intake  (g/week/rat) | 152.4 ± 7.1 174 ± 7.4 | 145.8 ± 3.6 146 ± 4.1 | 176.6 ± 6.8 157 ± 5.9 | 180.8 ± 5.5 190.2 ± 9 |
| Water Intake  (mL/week/rat) | 268.2 ± 8.6 286.6 ± 13.7 | 299.8 ± 12.8 288.6 ± 4.7 | 312.0 ± 9.1 265.8 ± 12.2 | 285.4 ± 11.3 296.8 ± 22.2 |
| Urine Output  (mL/rat) | 9.2 ± 0.9 8.8 ± 1.7 | 14.7 ± 1.8 12.4 ± 2.1 | 13.8 ± 2.5 15.6 ± 3.4 | 11 ± 0.9 14 ± 2.1 |

Data are presented as mean ± SE (n= 5 rats/group). Asterisk denotes statistical significance from the control (P <

0.05; unpaired t-test, Welch correction).

No significant changes in electrocardiogram endpoints (PR interval, heart rate, QRS complex and QTc interval) were observed in the control and the 0.05% groundwater-exposed groups at the specific time-points as shown in Figure S4.


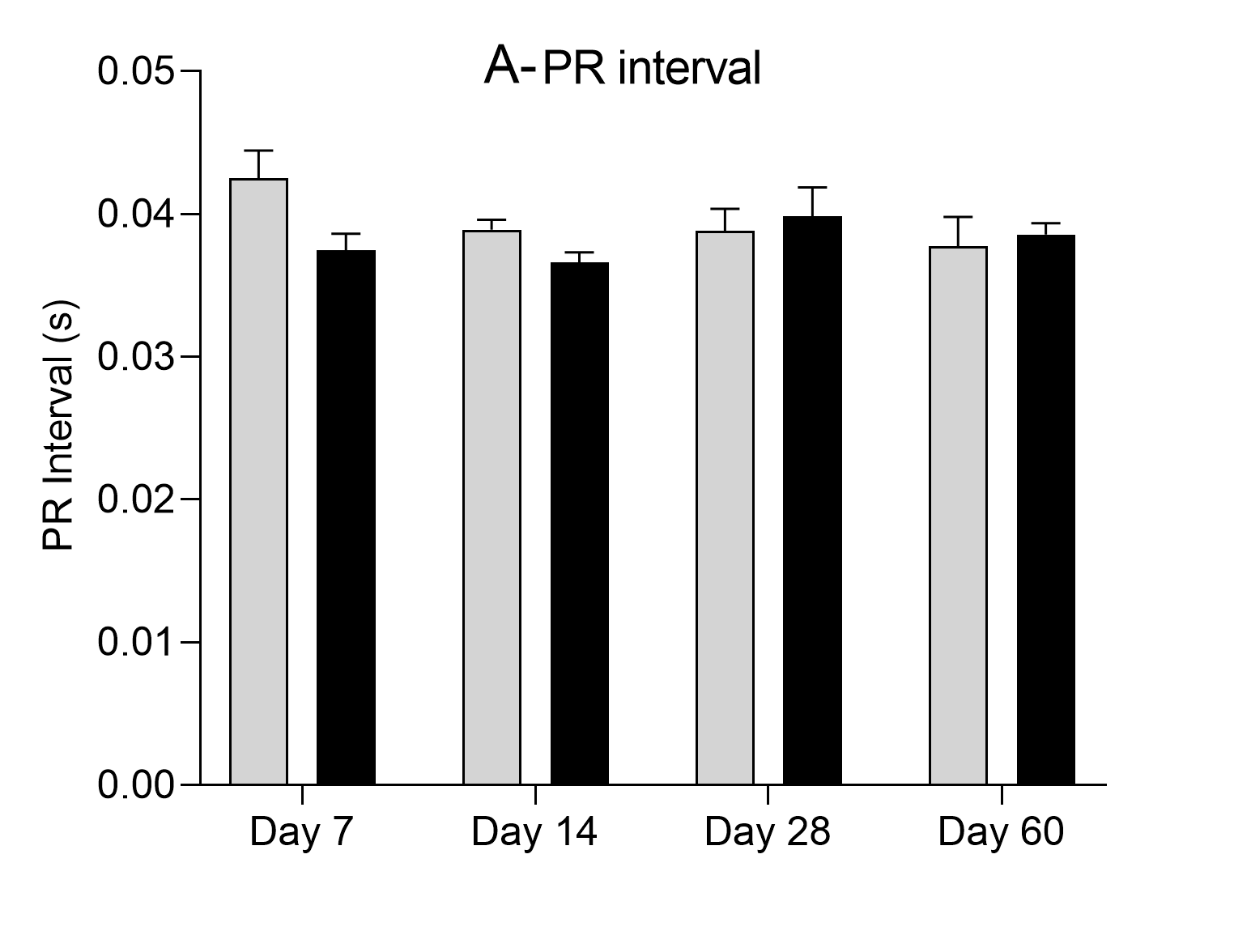

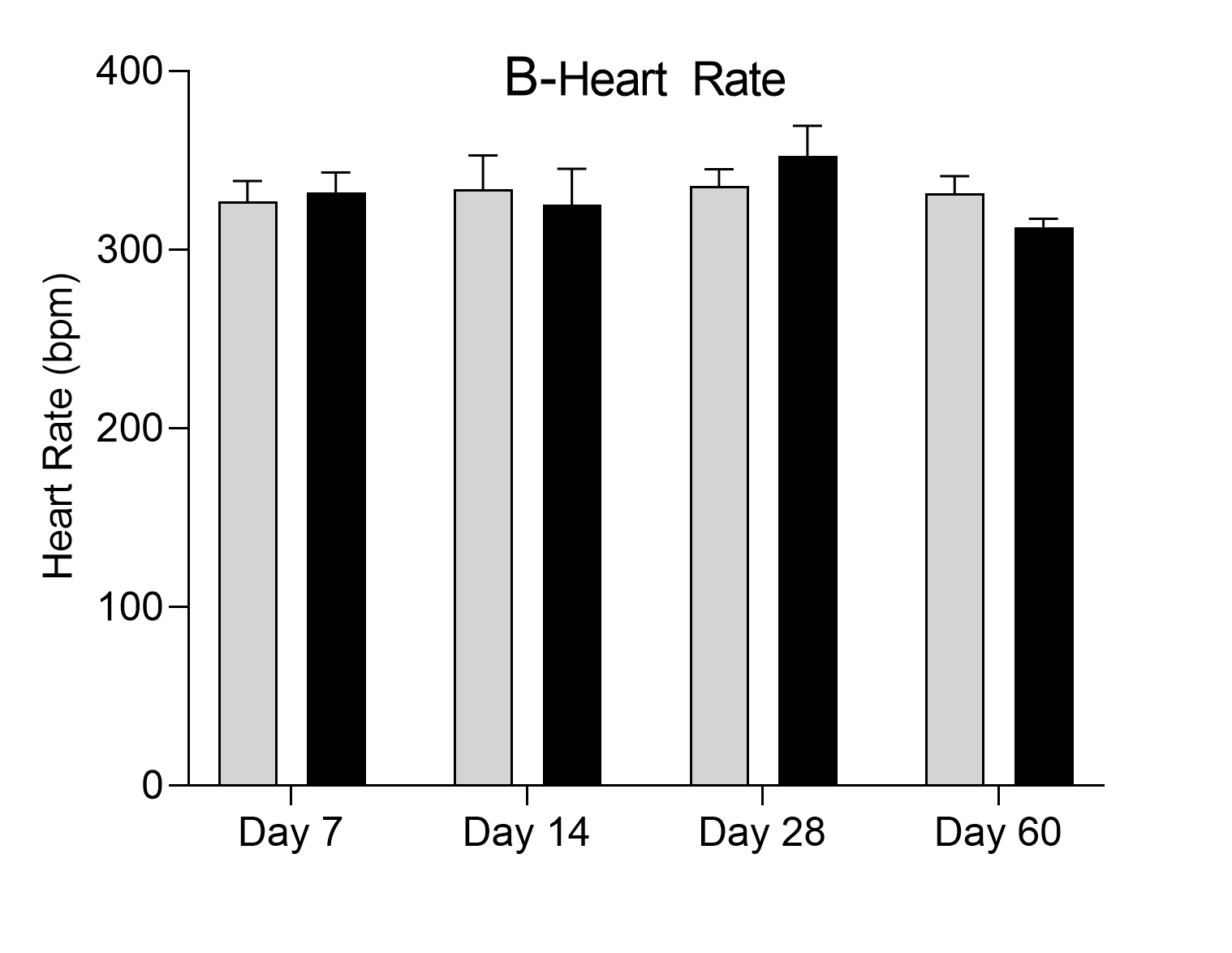

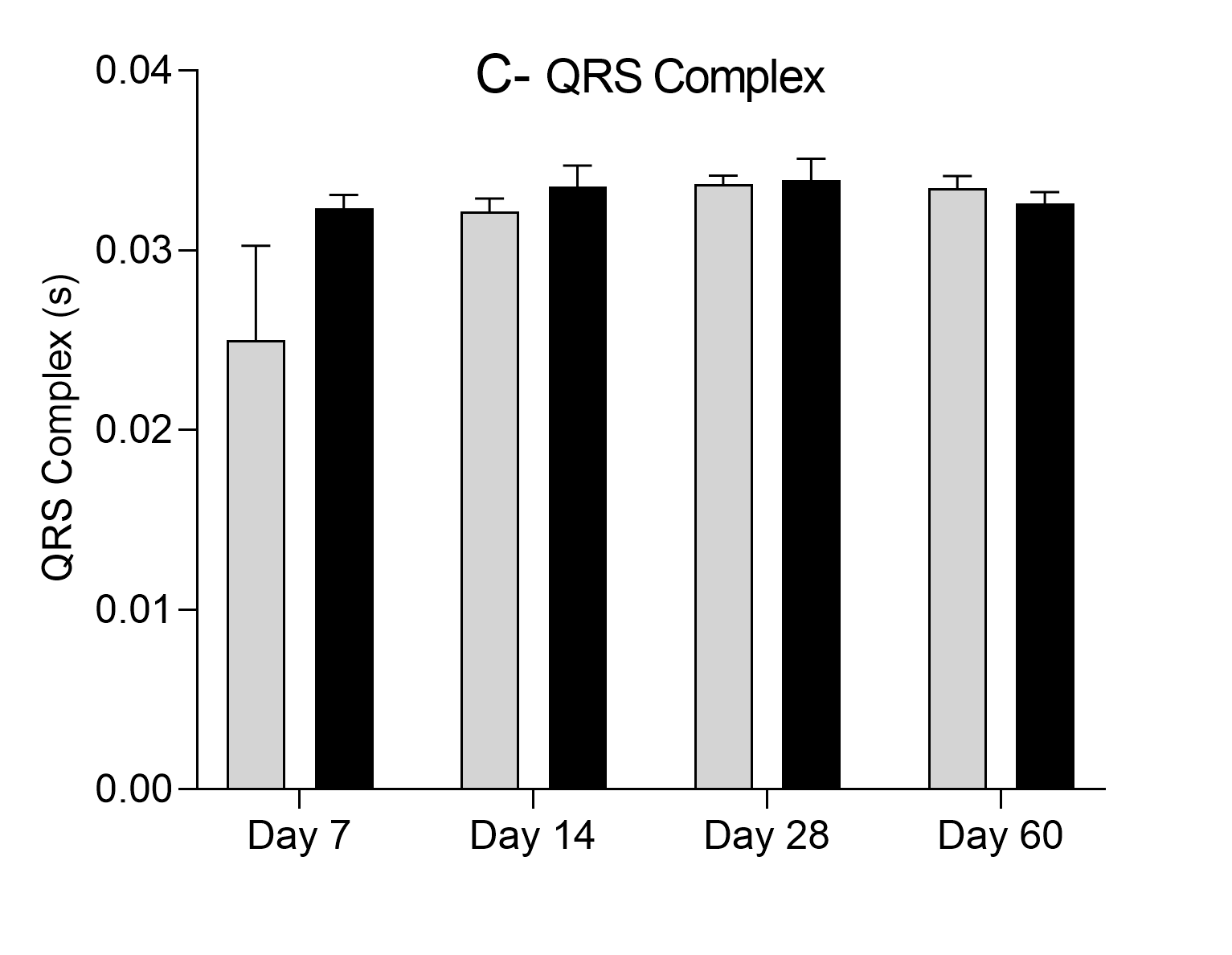

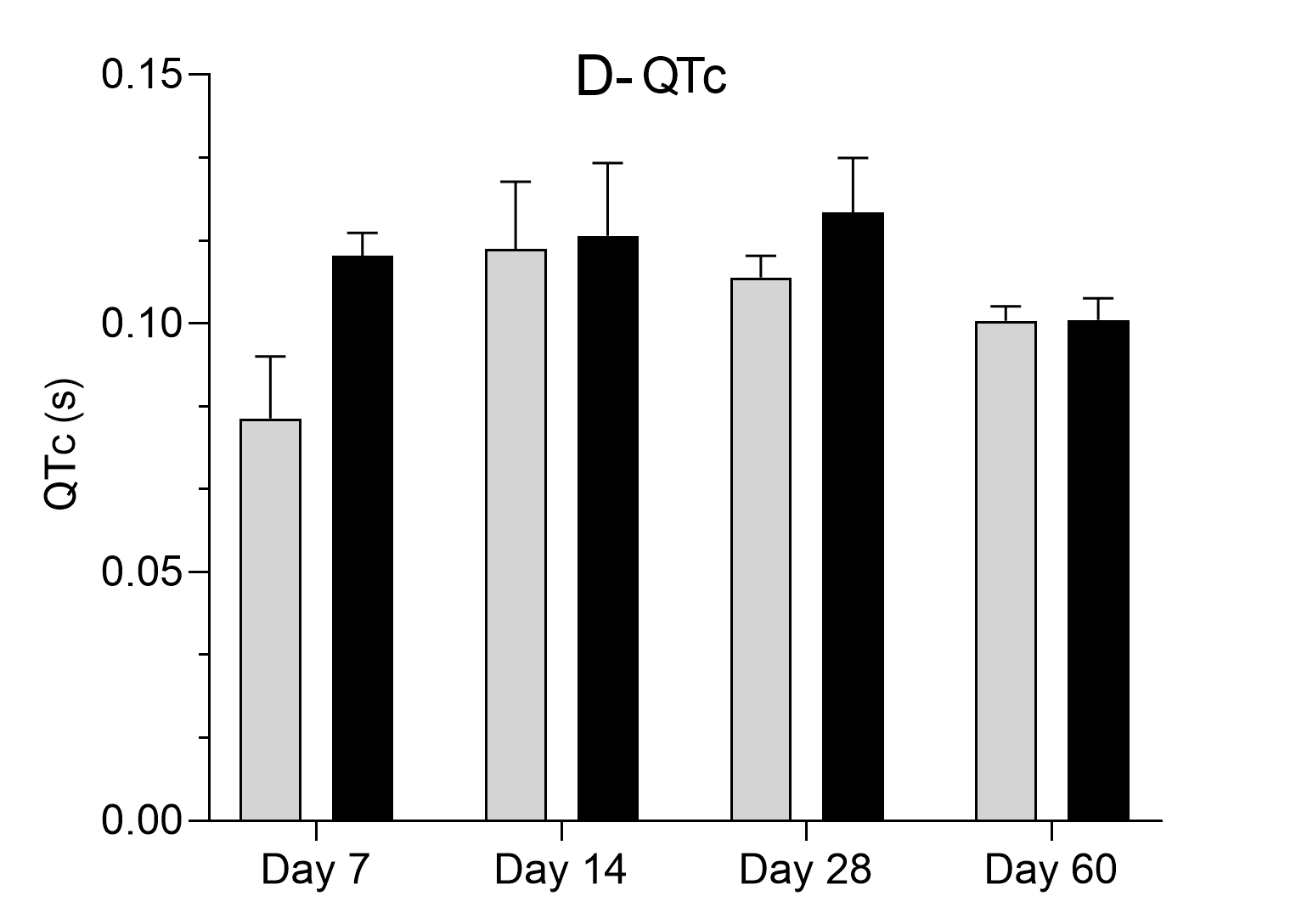


Figure S4 Cardiac electrical activity assessed using electrocardiogram interval analyses (A: PR Interval; B: Heart rate; C: QRS interval; and D: QTc duration) in male Sprague Dawley rats exposed daily to 0.05% v/v complex groundwater mixture (represented by black bars) or tap water (represented by grey bars) for specific time-points (7, 14, 28 and 60 days). Data are shown as mean ± standard error of the mean (n=5 control + 5 exposed/time-point). No statistically significant differences from the control were detected (P >0.05; unpaired t-test, Welch correction).
